# Large-Area Piezoelectric PVDF Fibers Fabricated by Near-Field Electrospinning with Multi-Spinneret Structures †

**DOI:** 10.3390/mi8040097

**Published:** 2017-03-24

**Authors:** Cheng-Tang Pan, Kuo-Chang Tsai, Shao-Yu Wang, Chung-Kun Yen, Yan-Liang Lin

**Affiliations:** Department of Mechanical and Electro-Mechanical Engineering and Institute of Medical Science and Technology, National Sun Yat-sen University, Kaohsiung 80424, Taiwan; pan@mem.nsysu.edu.tw (C.-T.P.); sywang1991@gmail.com (S.-Y.W.); alden0113@gmail.com (C.-K.Y.); 0912anson@gmail.com (Y.-L.L.)

**Keywords:** near-field electrospinning (NFES), multi-spinneret, piezoelectric fibers, polyvinylidene fluoride (PVDF)

## Abstract

In the study, we improved the near-field electrospinning (NFES) by multi-spinnerets with a cylindrical collector to fabricate a large area permanent piezoelectric of polyvinylidene fluoride (PVDF) fibers array. We designed multi-spinnerets by using printed circuit board (PCB) and drilled spinnerets on the solder balls. With different process parameters, we can obtain different diameters of PVDF fibers. By using the Taguchi method analysis, we found that the optimum sample of PVDF fiber arrays were manufactured by an electrical field of 1.6 × 10^7^ V/m. The cylindrical collector with high tangential velocity of 1779.9 mm/s and the heat treatment temperature of 65 °C for one hour. In addition, we used X-ray diffraction (XRD) and scanning electron microscopy (SEM) to analyze β-phase crystal quality and the surface character of PVDF fibers, respectively. From the observation of XRD, it revealed a high diffraction peak at 2θ = 20.6° of piezoelectric crystal β-phase structure. As PVDF solution with concentration of 18 wt % and the conductivity of 44.2 μS/cm was electrospun via NFES with multi-spinneret structure, we obtained a smooth manufacturing process. When the periodical tapping frequency was applied with 9 Hz, the maximum peak voltage of 86.9 mV was generated. In a cicada’s wing test, when the tapping frequency input was applied during 10–50 Hz, the maximum output voltage signals of 6.2 mV were generated.

## 1. Introduction

Electrospinning technology has been one of the common processing technologies in industrial production for microfibers since the 1990s. It was hard to collect polyvinylidene fluoride (PVDF) fibers orderly by the traditional electrospinning. Another issue was that it required high voltage. Therefore, how to reduce the operating voltage of electrospinning and to collect fibers orderly have become the focuses of the research on electrospinning in order to accelerate the production of electrospun fibers. The PVDF piezoelectric material has been extensively studied and used in sensor and biomedical domains for its flexibility, low price, high sensitivity, high piezoelectric coefficient and high biocompatibility. The PVDF can be made into film, bulk and tubular structures by means of extrusion, casting and die pressing. The PVDF piezoelectric material is extensively studied and used.

In comparison to the extensive studies and application of PVDF piezoelectric films [1], current research on PVDF piezoelectric fibers is scarce. In 2010, Pu et al. indicated that the piezoelectric strain coefficient d33 of PVDF piezoelectric fiber was −57.6 pm/V, and the PVDF piezoelectric strain coefficient d33 of piezoelectric film on the market was −25 pm/V [2]. The piezoelectric property of PVDF piezoelectric fiber is better than that of film, and it has greater potential in expandability in its applications, such as on micro/nano-sensors and generators [3]. The crystal of PVDF has three phases, including α, β, and γ. The content of β phase represents the piezoelectric strength of PVDF material; the γ phase is the mixed crystal phase of α phase and β phase, thus its piezoelectric property is in the next place [4,5]. When the PVDF with a high content of α phase was polarized by heating, high voltage, and stretching (or extrusion), then the dipole moments realigned from a disorder status to a single directional status; therefore, the crystal α phase is converted into β phase [6,7,8,9]. The static field electrospinning is the most convenient and fast method for producing PVDF piezoelectric fiber at present. In 1936, Formhal developed a technique for producing electrospun fibers and used the force of electric charges to make polymer material into fiber, which initiated the development of electrospun fiber [10]. In the 1960s, Taylor studied the Taylor cone [11] and jetting of polymer droplets in a high-voltage electrical field. When the electric charges accumulated in the high-voltage electrical field, and overcame its surface tension, then the droplet on the spinneret formed Taylor cone. Therefore, the polymer solution was ejected out of the conical tip and formed a long and thin polymer fiber. Under the mutual exclusion of electric charges and the effect of electrical field, the fiber is whipped and pulled violently, and deposited on the collector plate randomly. Thus, the collected fiber was disordered and not uni-directional. In 2006, Sun et al. proposed the near-field electrospinning (NFES) manufacturing process [12]. In the electrospinning process of NFES, the distance between needle head and electrode was reduced to millimeter scale, providing a better fiber deposition controllability. In comparison with far field electrospinning (FFES) and NEFS, FFES requires higher high voltage power supply to provide electrical field than NFES. The higher voltage will raise the safety risk and impact from operation of experiments’ conduct and safety to equipment and operators. In addition, the fiber collection of FFES is harder to collect orderly for its long distance than that of NEFS. Therefore, NFES has these two strengths more than FFES in terms of producing operation and fiber collection. In 2009, a direct-writing electrospinning technique was proposed to electrospin PVDF piezoelectric fibers [13,14]. The PVDF solution was placed in the needle cylinder, and the needle head was applied with high voltage. The PVDF powder is nonpolar α phase with random orientation of dipoles. The process of NFES applied strong electric fields (greater than 10^7^ V/m) on it. In addition, the stretching forces from cylindrical collector of the electrospinning process align dipoles in the fiber crystal. It makes the nonpolar α phase (random orientation of dipoles) to be transformed into the polar β phase. It is the NFES process that determines the polarity of the electrospun fiber. The proposed technique can reduce the spacing between needle head and collector plate from tens of centimeters to 1mm, and thus the required voltage is greatly reduced.

At present, how to produce microfibers orderly with good piezoelectric property in large quantities is a topical subject in the studies of electrospinning, and it also is a critical threshold for the commercial applications of the PVDF fibers. One of the methods to increase the production of electrospun fibers is to increase the quantity of needle heads [15,16]. However, the arrayed needle heads have a complex structure, which is a high price device, and they are prone to be blocked in the electrospinning process. The mutual interference between needle heads results in a disorderly deposit of the fibers. Therefore, the studies on needleless electrospinning came out. For example, using bubbles as triggers [17] or using conical wire coils [18] can multiply fibers output by means of using multiple jets, so as to increase the fibers production. Theron et al. used liquid surface perturbation of magnetic fluid to make multi-jet electrospinning as a method of needleless electrospinning [19]. Dosunmu et al. used gas pressure to push the polymer solution through the cylindrical porous foam surface to produce multiple jets, so as to attain high rate production [20]. Fuh et al. developed a needleless foam to be used for a large area electrospinning technique in order to overcome the limitation of electrospinning production output [21]. Multiple threads of fiber can be ejected at the same time, but it was not a continuous electrospinning process. Due to various sizes of foam pores not being uniform, the diameters of fibers were not uniform. The fibers have to be ejected momentarily by pressing the tweezers in each electrospinning process, so the fibers cannot be collected continuously and orderly. A modified electrospinning process with an auxiliary grounded electrode was developed to improve the production rate of nanofibers. This technique provides a novel route for electrospinning apparatus design and has potential for high-throughput nanofiber fabrication. The productivity was 7–10 times than that of traditional electrospinning [22]. In the study, we used Multi-spinneret Structures to raise the throughout. It had higher productivity than that of single-spinneret structure. For instance, the four-spinneret structure will produce four times throughout that from the single-spinneret structure. Fang et al. proposed needleless electrospun PVDF webs to enhance mechanical harvesting. It achieved higher productivity and better energy harvesting performance [23]. Persano et al. developed aligned arrays of nanofibers of poly(vinylidenefluoride-*co*-trifluoroethylene) to obtain rugged lightweight construction and ultra-high sensitivity of pressure sensors [24]. A novel technique was presented using needle-disks as spinneret to enhance nanofiber throughput and maintain high quality nanofiber. Unlike previous research of needleless electrospinning, needles on disks provide sites of jet initiation, showing an active and more controllable process of multiple jets generation [25]. A needle-disk electrode spinneret was designed through the combination of the point discharge concept and the merits of typical needleless electrospinning (disk as spinneret). Both the numerical simulation and experimental results showed that needle-disk electrospinning can produce competitive quality of nanofibers accompanied by enhanced throughput, compared with the traditional single-needle electrospinning method [26].

The above methods cannot collect multiple threads of electrospun fiber continuously, simultaneously, and orderly in a large area. Therefore, this study proposes a novel method, using needleless multi-spinneret structure in NFES to reduce the distance between collector and spinneret, and it also solves the interference problem between needle heads. The advantage is that the process has a higher production efficiency than a single needle process, and its collection method is continuous and ordered. According to X-ray diffraction (XRD) analysis, the obtained microfiber had good piezoelectric properties, and it can be the applications of sensors or energy harvesters.

## 2. Materials and Methods

### 2.1. Multi-Spinneret Structure

**Figure 1:**
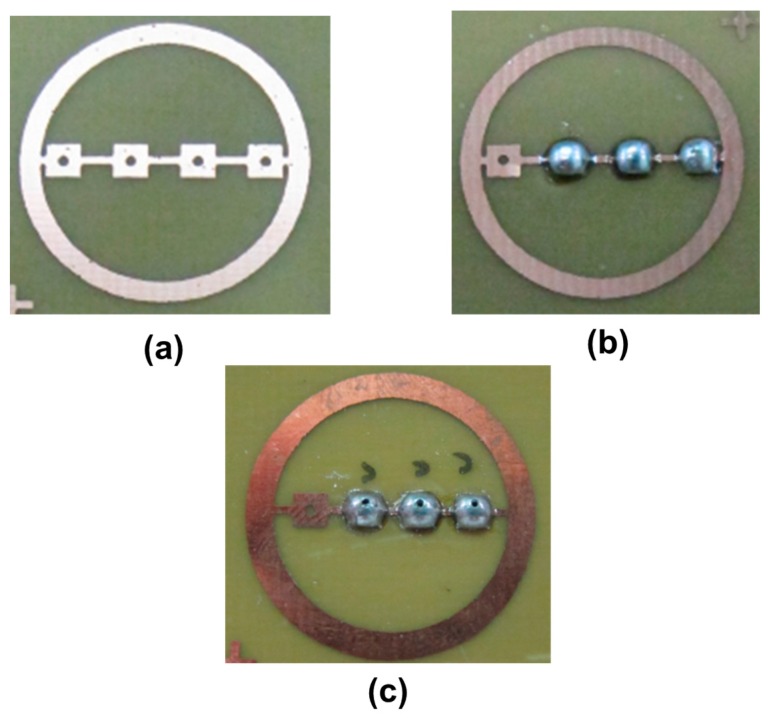
Multi-spinneret structure: (**a**) multi-spinneret circuit design; (**b**) multi-spinneret circuit design with solder balls; (**c**) drilled holes in solder balls.

This experiment used printed circuit board (PCB) to design different spinneret circuit structures to lay the metal conductive layer as shown in Figure 1a. Appropriate spinneret circuits can be designed for different applications in the future. The designed circuit board was soldered with solder balls structure (Figure 1b), and it was drilled by a precision driller to complete the multi-spinneret plate structure (Figure 1c).

When the innovative spinneret plate structure was completed, it was mounted on the self-made fixture for a multi-spinneret structure and chamber body. There was a flow channel inlet for a PVDF solution. Considering the closure between fixture structure and spinneret plate, there was an oil seal to prevent the PVDF solution leaking from flowing through hairline. There was an isobaric space for a PVDF solution chamber body, and the precision flow control pump was used at an injection rate of 0.007 mL/min to make the electrospinning more continuous and smooth. When the PVDF flew into the PVDF solution chamber, this chamber body had a good sealing characteristic to ensure these spinnerets with equal pressure. The multi-spinneret system did not have to set up additional complex flow channels or pipelines, so it is convenient for its extensive application in the future.

### 2.2. Manufacturing Process of Multi-Spinneret Structure

**Figure 2:**
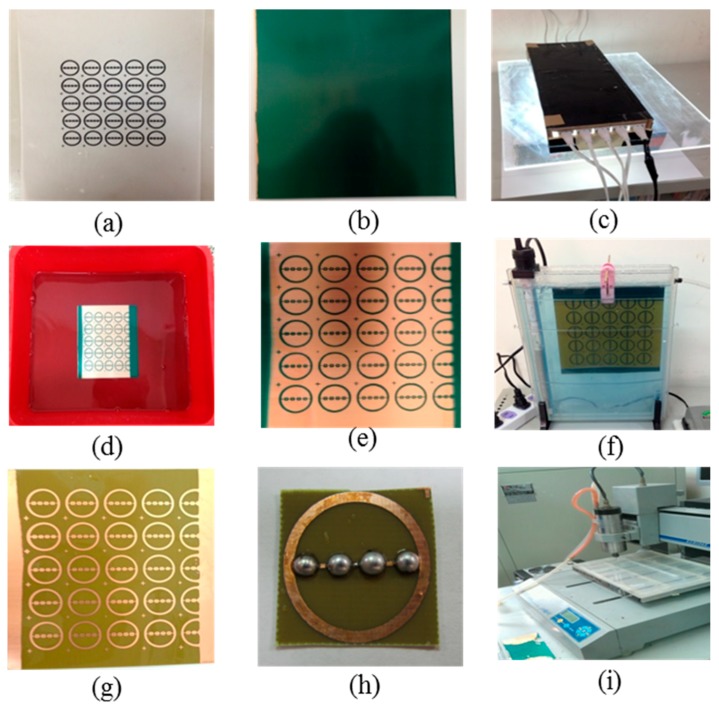
Multi-spinneret structure manufacturing process: (**a**) mask design; (**b**) duplicate the mask pattern to light-sensitive circuit board; (**c**) exposure; (**d**) development; (**e**) inspection; (**f**) etching; (**g**) film remove; (**h**) solder balls; (**i**) drill hole in solder balls.

First, the mask of the multi-spinneret plate was designed. The pattern was drawn by AutoCAD (see Figure 2a). Then, the mask pattern was copied onto the photosensitive circuit board, and it finished cutting. The photosensitive circuit board (sensitive coating thickness: 1.6 mm; copper foil thickness: 35 μm; board thickness: 0.6 mm) was taken out (see Figure 2b), then wrapped in light-tight paper, and cut by saw blades to meet the required size. The sawdust was cleaned up and the left photosensitive circuit board was put in a bag and placed in a cool place. Afterwards, the transparent or translucent original was aligned to the photosensitive plate, and it was covered with a transparent glass. A 10–20 W daylight desk lamp was placed 5 ± 1 cm above exposing for 7 min (see Figure 2c). The development was carried out after exposure. The developing agent was diluted 20 times with water to form developing solution. The exposed sensitive plate was placed in the developing solution with surface upward (see Figure 2d). After about 5 s, some green particles emerged. The container was jiggled at intervals of several seconds to disperse the particles, until the lines were very clear and there were no particles emerging. Then, the plate was washed with water and dried. Any scratch or imperfect surface was repaired or scraped by a knife to ensure it is in a good status; the integrity and clearness of the circuit design must be inspected as well (see Figure 2e). Then, the plate was soaked in ferric chloride solution for etching. First, the ferric chloride solution was poured into a plastic container (do not use metalwork; see Figure 2f), and the sensitive plate was placed in immersion and the film surface was upward. The etching process did not stop until the non-circuit copper foil was completely clean (see Figure 2g); meanwhile, the container was kept jiggling at intervals. The appropriate solution temperature was 20–50 °C. The plate was washed with water and dried after etching. The photosensitive film was wiped down with alcohol. Meanwhile, the photosensitive film can play the role of protective layer for copper foil. The solder ball was soldered in designed positions on the photosensitive circuit board. The final step was to drill the solder balls (see Figure 2h). We used a precision machining driller to drill spinnerets in the center of solder balls. Thus, all of the manufacturing processes were completed (see Figure 2i).

## 3. Results and Discussion

### 3.1. Near-Field Electrospinning (NFES) Polyvinylidene Fluoride (PVDF) Fiber with a Hollow Cylindrical Collector Process by Multi-Spinneret Structure

**Figure 3:**
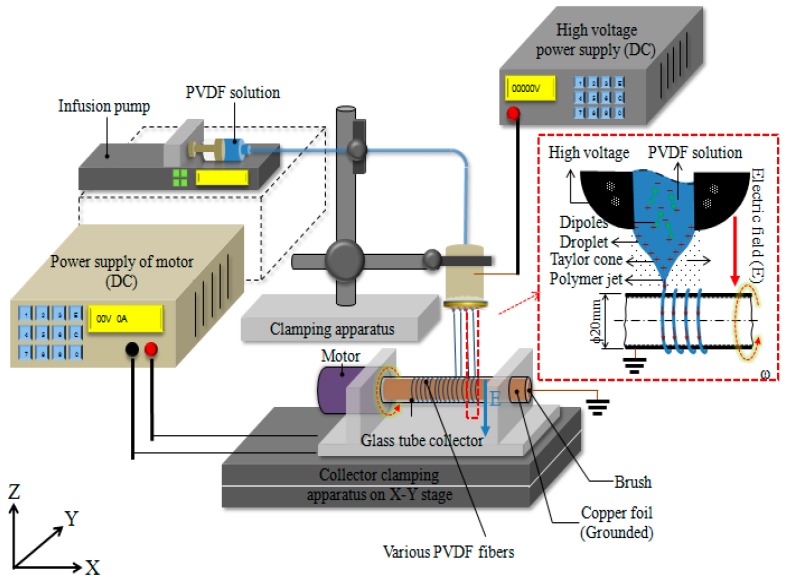
Equipment of polyvinylidene fluoride (PVDF) near-field electrospinning (NFES) with multi-spinneret structure cylindrical collector.

In this study, NFES PVDF fibers by multi-spinneret with a hollow cylindrical collector system, as shown in Figure 3. We developed the cylindrical collector, with a hollow glass tube, in order to collect the fibers orderly and continuously as Figure 3. Therefore, the method produced PVDF fibers in alignment, which also can be applied to produce a smart patch. It is based on an NFES concept to create multi-spinneret structure with a hollow cylindrical collector to collect a large area of PVDF fibers possessing good piezoelectric properties. In this study, a multi-spinneret structure was made by a Printed Circuit Board (PCB) design and solder balls. We researched improving stability of the process with different multi-spinneret structure by means of the Taguchi method. The factors included electrical fields (1.2 × 10^7^–1.6 × 10^7^ V/m), tangential speeds of cylindrical collector (1361.1–1779.9 mm/s; 1300–1700 rpm), and heat treatment temperatures (50–80 °C lasting for 1–3 h). By means of the analysis to find each parameter contribution degree, the follow-up analyses were carried out based on the optimized fibers, including voltage testing and cicada wing testing for the application of bionic sensors.

### 3.2. Measurement of PVDF Solution Concentration and Conductivity

In the experiment, the PVDF solution had different conductivities at different concentrations. The conductivity is a measurement value that indicates its ability to transmit current for this substance. In addition, the conductivity is a key factor of determining whether the PVDF solution facilitates the electrospinning process. This experiment attempted to find the concentration of PVDF solution possessing optimal conductivity by means of discussing the conductivities of PVDF solution in different concentrations, as shown in Table 1. The source of materials: PVDF powder (*M*_w_ = 534,000, Aldrich^©^, St. Louis, MO, USA), Acetone, Dimethyl sulfoxide (DMSO) and surfactant (fluorinated surfactant manufactured by DuPont^©^, Nevada, IO, USA). In order to dissolve PVDF in solution, we mixed the following two parts: Part A—we poured and distributed PVDF powder in Acetone and then stirred it with heat by magnetic stirrer on a heater about 30 min to make it uniform; Part B—we poured surfactant in DMSO and then stirred it with heat by a magnetic stirrer on a heater about 30 min to make it uniform. For the mixture of Part A and Part B, we poured Part A in Part B and then stirred it with heat by magnetic stirrer on a heater about 60 min to make it uniform.

**Figure 4:**
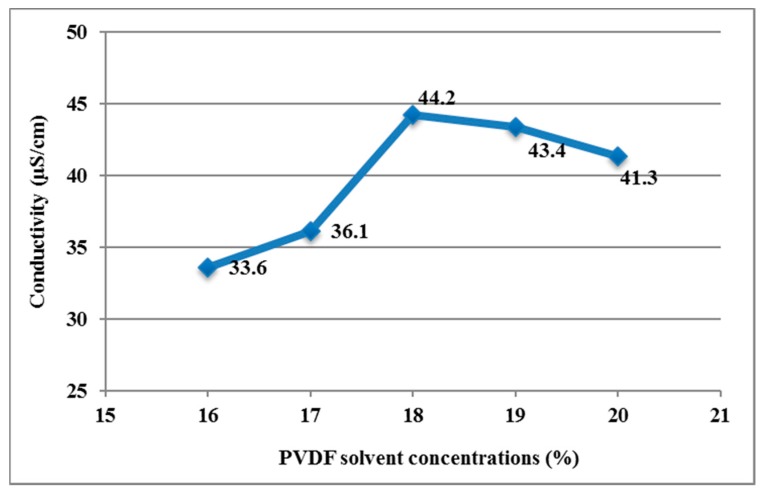
Relationship between conductivities and concentrations of PVDF solutions.

We use Orion^©^ model 150A plus conductivity meter (Thermo Electron Corporation, Beverly, MA, USA). We put the sensor end into the PVDF solution, and then it showed the reading of the solution. Figure 4 shows the relationship between concentrations and conductivities of PVDF solution. The results show that the conductivity of PVDF solution in weight percentage of 18 wt % was 44.2 µS/cm, which was the highest in the range of 16 to 20 wt %. In other words, when the other parameters were fixed, the PVDF solution in weight percentage of 18 wt % was the easiest concentration to accumulate electric charges on the droplet to form Taylor cone droplets and to eject PVDF piezoelectric fibers. Therefore, this study used PVDF solution in weight percentage of 18 wt % to carry out NEFS with multi-spinneret structure.

### 3.3. Influence of Solder Balls Size in NFES with Multi-Spinneret Structure

The experiment used NFES with multi-spinneret structure to produce PVDF piezoelectric fibers. Considering the influence of solder ball size on the multi-spinneret structure of NFES process, three sizes of solder balls were designed. The largest solder ball was designed with a height of 2.5 mm; the medium solder ball with a height of 1.5 mm; and the smallest solder ball with a height of 1.0 mm. Another one was zero solder ball (0 mm of radius; without the ball). Therefore, there were totally four sizes of spinnerets in this experiment. It was also equipped with a cylindrical collecting device. The experimental parameters were fixed: *XY* dual-axis digital control platform moves collector plate was 3 mm/s; the rotation speed of cylinder collector was 1500 rpm; the electrical field was 1.6 × 10^7^ V/m; and the diameter of spinneret was fixed at 0.5 mm. When the PVDF solution was infused by a pump at 0.007 mL/min, the charges accumulated in the PVDF solution and then the spinnerets’ jetted fibers.

**Figure 5:**
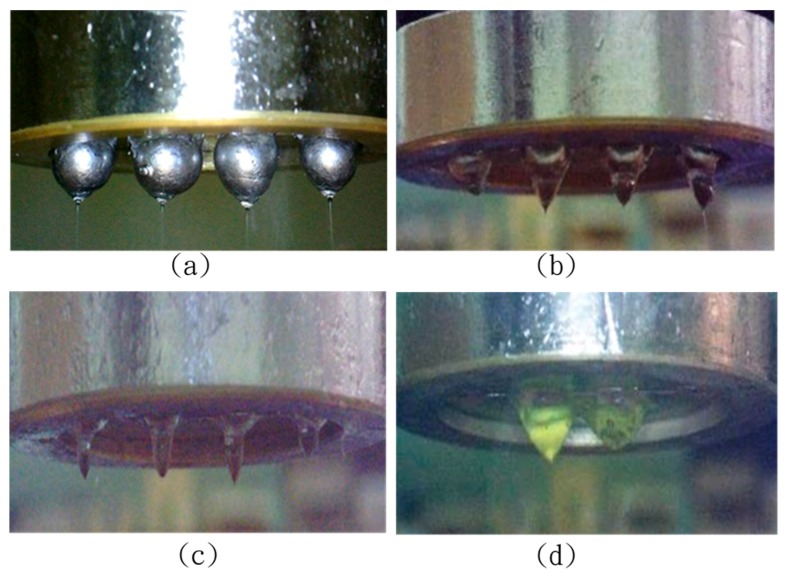
Large, medium, small, and no solder ball spinnerets and their droplets: (**a**) droplet height: 0.9 mm; (**b**) droplet height: 1.4 mm; (**c**) droplet height: 2.5 mm; (**d**) droplet height: 3.2 mm.

In the experiment, four sizes of solder balls (large, medium, small, and zero) were soldered on the multi-spinneret structures. Then, the experiments of NFES with multi-spinneret structures were conducted. The results show that all the jetted fibers of the multi-spinneret structures were interfered by that zero solder ball. When the PVDF solution jetted through spinnerets, they were interfered by the adjacent spinnerets mutually, as PVDF solution itself had a certain consistency. The impact of the interference even came from all spinnerets. This resulted in large droplets eventually, which was a bad condition in this study. In the experiment, the largest solder ball surface accumulated enough electric charges when the PVDF droplet height was 0.9 mm. It broke through the PVDF solution surface tension, and the PVDF solution jetted fiber stably, as shown in Figure 5a. The follow-up droplet heights in descending order were medium solder ball, small solder ball and zero solder ball, as shown in Figure 5b–d. The larger solder balls were the shorter droplets’ height, and then the manufacturing processes were more stable in the shorter droplets’ cases.

### 3.4. Influence on the Fibers Diameter from Electrical Field

**Figure 6:**
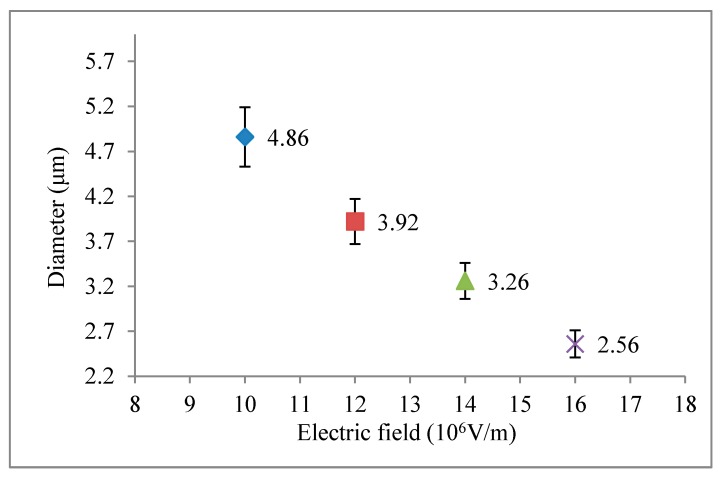
Relationship between electrical fields and diameters of fibers.

**Figure 7:**
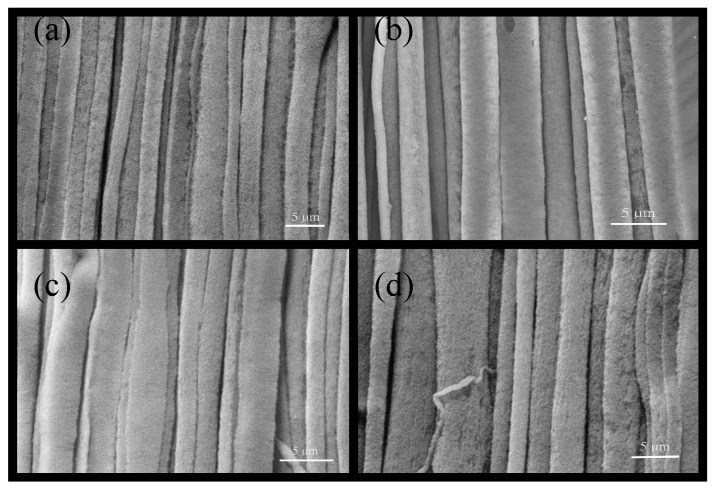
Scanning electron microscopy (SEM) images of electrospinning fibers in different electrical fields: (**a**) 16 × 10^6^ V/m; (**b**) 14 × 10^6^ V/m; (**c**) 12 × 10^6^ V/m; (**d**) 10 × 10^6^ V/m.

To explore the influence from driving electrical fields on the diameter of fibers, the experimental parameters were fixed: the rotation speed of cylinder was 1200 rpm; PVDF solution was 18 wt %; moving speed of *XY* dual-axis digital control platform was 3 mm/s. The only change was driving electrical field: 1.0 × 10^7^, 1.2 × 10^7^, 1.4 × 10^7^, and 1.6 × 10^7^ V/m. When other experimental parameters were fixed, the driving voltage in the electrospinning process controlled the electrostatic force via the electrical field. It will influence Taylor cone for electrospinning. As the electrical field is stronger, the electrostatic force will be stronger and the force to attract the solution will also be larger. Therefore, the fiber diameter will be thinner. The relationship between electrical fields and diameters of fibers is shown in Figure 6. According to scanning electron microscopy (SEM) observation of the electrospinning fibers, we can find that the diameters of fibers with respect to the electrical fields are: the diameters of fibers in the electrical field 1.6 × 10^7^ V/m were smaller and more even; the diameters of fibers with the electrical field 1.0 × 10^7^ V/m were larger and less even. Thus, the stronger the electrical field was, the more even the diameters of fibers will be. The SEM images show in Figure 7.

### 3.5. Analysis of NFES with Multi-Spinneret Structure by Taguchi Method

This section used the theory of Taguchi methods to design optimal parameters for PVDF piezoelectric fibers fabricating via NFES with multi-spinneret structure. First, nine groups of experimental parameters were planned by L9 orthogonal array in the experiment. The influence on the piezoelectric properties by the parameters of NFES with multi-spinneret structure were estimated by XRD (Bruker AXS GmbH Bruker D8, Karlsruhe, Germany) and voltage signals analyses. In the Taguchi method, the response table is obtained from signal-to-noise (*S*/*N*) ratio to determine the optimal process parameters. The contribution degree and analysis of variance (ANOVA) can be calculated. Then, by means of overall quality analyses to determine the optimal parameters for PVDF piezoelectric fibers fabricating process for NFES with multi-spinneret structure. Nine groups of experimental parameters were determined by the L9 orthogonal array of Taguchi method, as shown in Table 2. The parameters of electrical field intensity, hollow cylindrical collector rotation speed, fiber heat treatment temperature, and retention time were changed in the experiments. If those parameters had no interaction, they were used to analyze the conversion between α phase and β phase for PVDF piezoelectric fibers.

**Figure 8:**
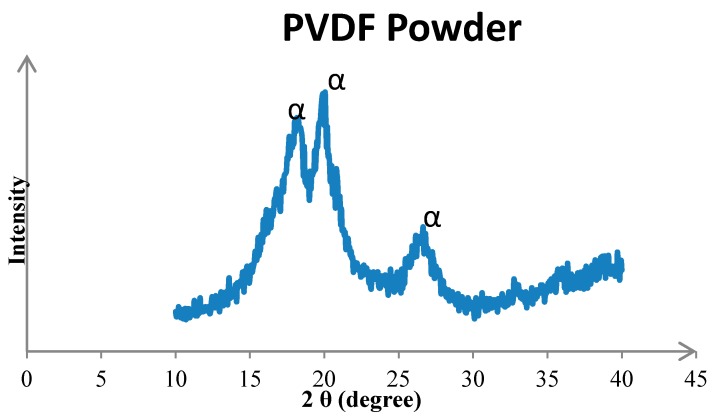
X-ray diffraction (XRD) chart of PVDF powder.

The crystal phases of PVDF included α, β and γ. The α phase crystal is most common in the unpolarized PVDF powder and film, and β phase is the most important crystalline phase to account for piezoelectric property. Thus, the α phase crystal is converted into β phase crystal by polarization. The dipole moments of β phase crystal were polarized directionally, and the dipole moments arranged in the same direction to make the PVDF possessing piezoelectric property. Therefore, the content of β phase represents the piezoelectric strength of PVDF. Three major angles of α phase crystal in PVDF were 18.4°, 20.1°, and 26.8°. The β phase major angle was 20.6°, which is major crystal phase for piezoelectric property. According to observation, in which the crystal lattice of the original PVDF powder was α phase crystal, without piezoelectric property before the manufacturing process (see Figure 8).

**Figure 9:**
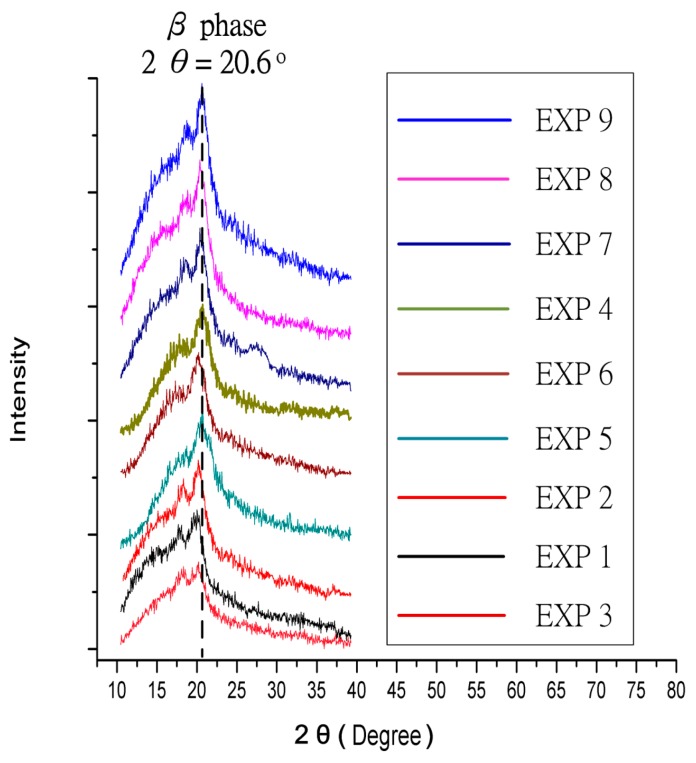
XRD charts of nine groups with different parameters using the Taguchi method.

This experiment of the Taguchi method used XRD measurement and voltage measurement for mutual validation. The larger-the-better analysis of the Taguchi method was carried out with measuring the voltage output. The different electrical fields and cylindrical collector speeds were delivered to observe the process of NFES with multi-spinneret structure. The fibers were put in the oven for heat treatment before they were dried completely. In order to investigate the influence of the crystal structure in piezoelectric fibers from changing process parameters, the crystal inside the PVDF fiber was converted from a common α phase crystal without a piezoelectric property into β phase crystal, so that the PVDF fibers were able to have a piezoelectric property. The PVDF powder needs to be grounded up, then filtered by a 325 mesh filter before measuring, and then used a Bruker D8 X-ray powder diffractometer to measure XRD data. The X-ray can inspect the transformation of β phase in PVDF fibers. By means of this X-ray powder diffractometer inspection, we can evaluate that the impacts of process parameters to the transformation of β phase in PVDF fibers. The operation specifications of the Bruker D8 X-ray powder diffractometer: range of 2θ: 15° to 40°, scanning interval of angle: 0.05°, and scanning interval of angle: 3 s. In the experiment, we changed some process parameters, including electrical field intensity, cylindrical collector rotation speed, fiber heat treatment temperature and retention time, in order to analyse the conversion between α phase and β phase inside PVDF fibers by means of the analysis to find optimal parameters for piezoelectric fibers. Based on the XRD measurement results, the strengths of β phase were arranged in descending order as shown in Figure 9.

### 3.6. Voltage Analysis of Nine Groups of PVDF Piezoelectric Fibers by the Taguchi Method

**Figure 10:**
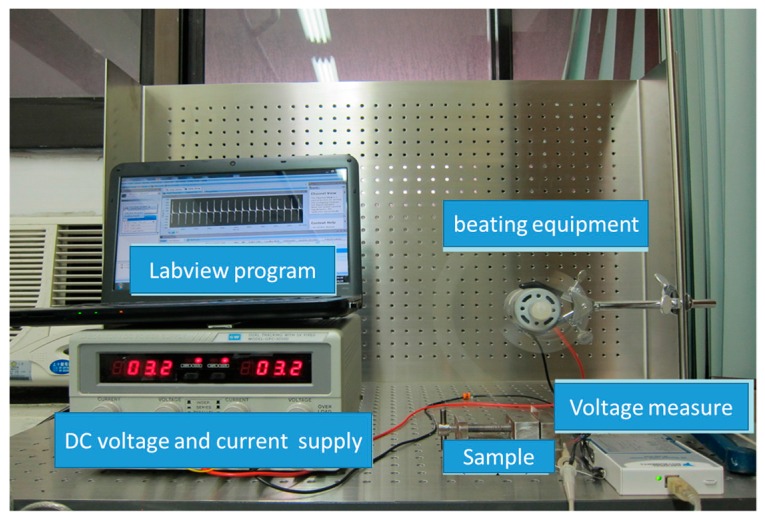
Beating test equipment for voltage measurement.

To calculate the *S*/*N* table and contribution ratio of various parameters to PVDF fibers by NFES with the multi-spinneret structure, the nine experiments of fibers were conducted with a beating test for voltage measurement (Figure 10). The voltage magnitude of beating test results were quantified and then compared with the XRD measurement results, in order to determine the piezoelectric strength. The ordered PVDF fibers were taken from the cylindrical collector and placed on copper electrodes and then coated with silver gel, so that the PVDF fibers and copper electrodes can be bonded well. Then, it was packed with PE membrane. The spacing between two electrodes was 0.5 mm in the copper parallel electrodes. Each group was tested three times, and then the average of the three times values was taken.

According to the nine groups of Taguchi method XRD chart (see in Figure 9), the NFES with multi-spinneret structure PVDF process was conducive to crystallize β phase crystallization. Thus, original α phase crystal was decreased apparently, and the originally nonexistent β phase of crystallization emerged. Therefore, the NFES with multi-spinneret structure was helpful to form the piezoelectric fibers of PVDF.

### 3.7. Parameter Contribution Degree

**Figure 11:**
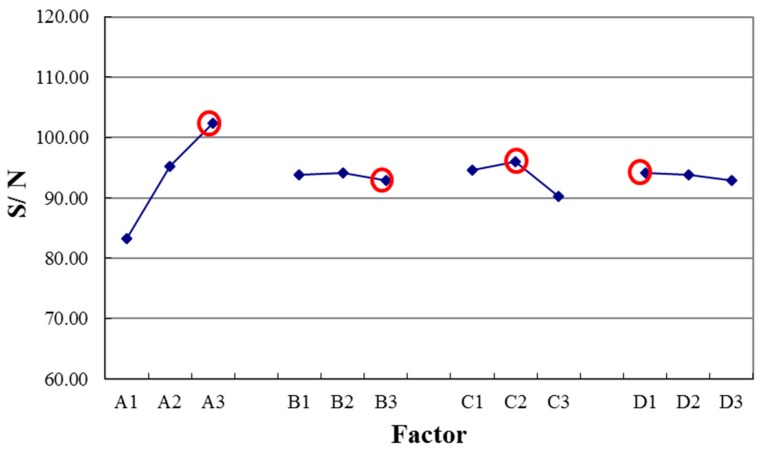
Signal-to-noise (*S*/*N*) ratio comparison chart of electric field, speed of cylindrical collector, heat treatment temperature, and temperature holding time.

According to the above nine groups of voltage output results, the study observed the response values of *S*/*N* ratio and quality characteristic factors at various factors with various levels. We can get sensitivities of the various factors by subtracting variations of the various parameters from each other. When the factor was at a higher sensitivity, the factor was more important, and vice versa. According to the *S*/*N* ratio factor response diagram of PVDF piezoelectric fiber voltage output (see in Figure 11), the electrical field was the maximum control factor of influencing the voltage output of piezoelectric PVDF fibers. The other factors following it in descending order were heating temperature, the rotation speed of the cylindrical collector, and the retention time. Table 3 shows the contribution of each process parameter of PVDF contributes to piezoelectric effect output voltage of PVDF. As shown in Table 3, the highest contribution came from electrical field (90.23%). The other factors followed with it in descending order were heating temperature (9%), rotation speed of cylindrical collector (0.40%), and retention time (0.37%).

### 3.8. Voltage Test of PVDF Fibers at Different Slap Frequencies

**Figure 12:**
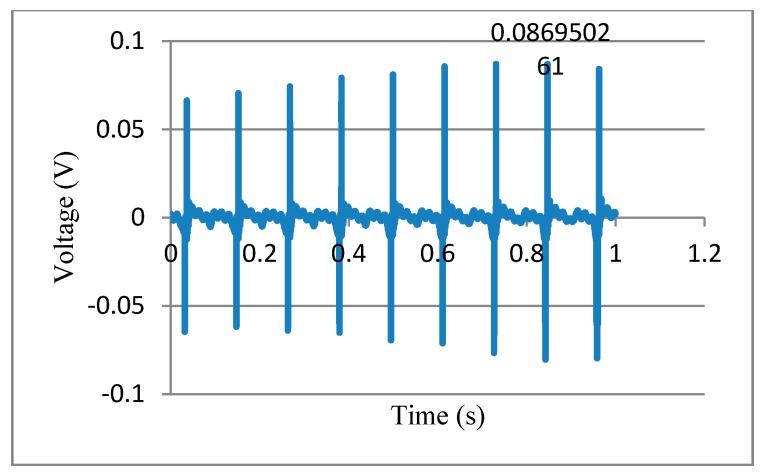
Energy harvesting voltage measurement (9 Hz).

In this experiment, we analyzed optimizing fiber by the Taguchi method, using 18 wt % PVDF solution with best conductivity; the electrical field was 1.6 × 107 V/m; the rotation speed of glass cylindrical collector was 1700 rpm; and heating temperature was 65 °C for one hour. Using 0.1 m slapping rod to slap PVDF fibers specimen, the specimen had about 1000 PVDF piezoelectric fibers with 1 cm length and about 3 μm diameter. The spacing of parallel electrodes was 0.5 mm. Measuring PVDF piezoelectric specimen relation between different slapping frequency and output voltage, the measurement equipment is shown in Figure 10. In the experiment, we used parallel electrodes to be energy harvesting devices, and the specimen was fixed to one end of the cantilever state. Then, we used a slapping device that was made of the rotating motor for slapping. The capacitance effect happened between two parallel electrodes spacing when the specimen slapped, and voltage signal was generated (see Figure 12).

### 3.9. Cicada’s Wing of Experimental Bionic Voltage Signal Measurements

**Figure 13:**
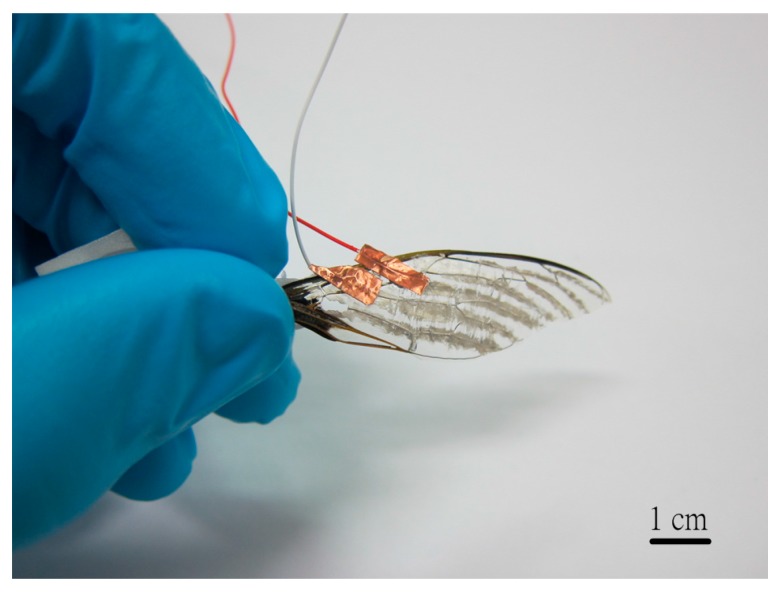
Sample for vibration test of cicada’s wing.

In the experiment, we used the wing of Formosan Bear Cicada (scientific name: *Cryptotympana holsti*) to test if it is possible to apply PVDF fibers on insect sensing. It is reasonable to estimate that the flapping frequency of cicada is between 10–50 Hz [27]. We coated silver gel as electrodes in the bigger skeleton of a cicada’s wing. If we attached wires or electrodes on a cicada’s wing, it will increase the stiffness of a cicada’s wing in flapping to result in more measured errors. Thus, we replaced copper electrodes or wires by silver gel as electrodes to test voltage harvesting in a cicada wing vibration. The vibration test of a cicada’s wing specimen is shown in Figure 13. The direction of silver gel coating referred to the real-life vibration of a cicada’s wing when it is flying.

**Figure 14:**
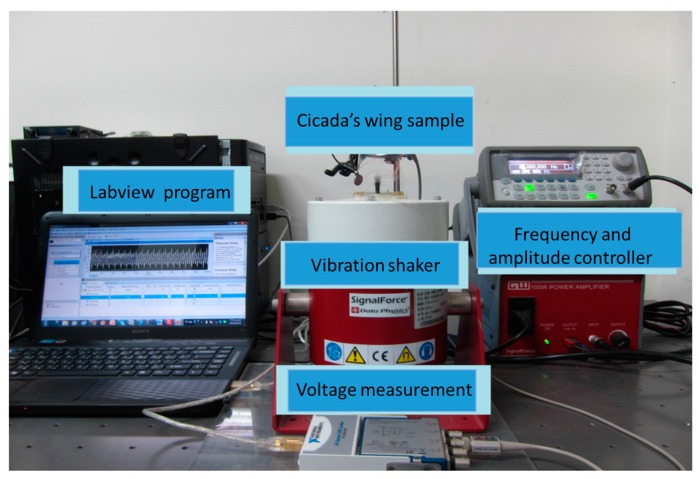
Voltage measurement equipment for a cicada’s wing.

**Figure 15:**
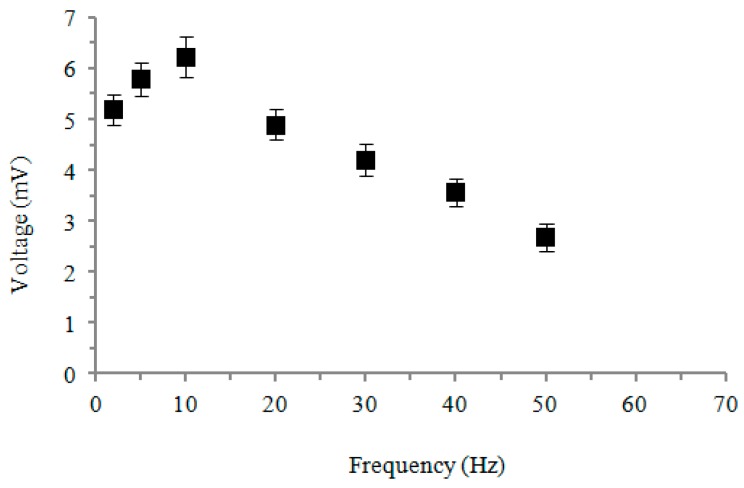
Relationship between vibration frequency of a cicada’s wing and response voltage.

The equipment for a cicada’s wing vibration test is showed in Figure 14, and we applied vibration at 10–50 Hz to generate the output voltage as shown in Figure 15. A maximum peak voltage of 6.2 mV was generated. The process was successfully demonstrated in which NFES with the multi-spinneret structure PVDF fiber process can be applied on bionic sensing in the future.

## 4. Conclusions

The study proposes a new process by means of NFES with a multi-spinneret structure and a cylindrical collector to fabricate PVDF fibers orderly in a large area. After continuous improving on multi-spinneret component, multiple threads of PVDF fibers can be ejected at the same time, and it was combined with a hollow cylindrical collector and an *XY* dual-axis digital control platform to produce PVDF fibers quickly with good piezoelectric properties. By discussing the conductivity about PVDF with different concentrations between 16–20 wt %, we found 18 wt % PVDF solution had the best conductivity of 44.2 μS/cm. In the experiment of multi-spinneret design with different heights of solder balls on NFES, we found that higher solder balls that more easily accumulated Coulomb force and PVDF fibers can be jetted stably with a droplet height of 0.9 mm. In the process of NFES with a multi-spinneret structure and a hollow cylindrical collector, when the speed of the cylindrical collector increased (in the range of tangential velocity 942.3 to 1884.6 mm/s) and the electrical field (in the range of 1.0 × 10^7^ to 1.6 × 10^7^ V/m) increased, and then the diameters of PVDF fibers will be smaller. Considering the influences from the electrical field, speed of cylindrical collector, heat treatment temperature, and retention time on voltage output of PVDF piezoelectric fibers, the result shows that the contribution of the electrical field was 90.23%, which was the most critical factor. The second largest contribution was heat treatment temperature (9%). The third largest contribution was rotational speed of the cylindrical collector (0.40%). The least contribution was retention time (0.37%). The PVDF fibers with the Taguchi method optimizing parameters will produce the voltage output up to 86.9 mV at the flop frequency of 9 Hz. In the bionic oscillation experiment, we successfully demonstrated that the fibers were placed on a cicada’s wing to produce obvious voltage output. It can produce the maximum voltage output of 6.2 mV at the frequency of 10 Hz.

## Figures and Tables

**Table 1:** Formula of polyvinylidene fluoride (PVDF) solution with different concentration. DMSO: dimethyl sulfoxide.

PVDF	PVDF	Solvent (DMSO:Acetone)		Surfactant
Weight Percent (PVDF/Solvent)	Weight (g)	DMSO (g)	Acetone (g)	Weight (g)
16 wt %	0.80	2.5	2.5	0.2
17 wt %	0.85	2.5	2.5	0.2
18 wt %	0.90	2.5	2.5	0.2
19 wt %	0.95	2.5	2.5	0.2
20 wt %	1.00	2.5	2.5	0.2

**Table 2:** Parameters in Taguchi L9 orthogonal table.

EXP	Electrical Field (V/m)	Cylindrical Collector Speed (rpm)	Fiber Heat Treatment Temperature (°C)	Temperature Holding Time
**1**	1.2 × 10^7^	1300	50	1 h
**2**	1.2 × 10^7^	1500	65	2 h
**3**	1.2 × 10^7^	1700	80	3 h
**4**	1.4 × 10^7^	1300	65	3 h
**5**	1.4 × 10^7^	1500	80	1 h
**6**	1.4 × 10^7^	1700	50	2 h
**7**	1.6 × 10^7^	1300	80	2 h
**8**	1.6 × 10^7^	1500	50	3 h
**9**	1.6 × 10^7^	1700	65	1 h

**Table 3:** The parameters with the Taguchi method.

The Contribution of all Parameters				
Factors	Degree of Freedom	Standard Deviation(*S*)	Variation (*V*)	Contribution (%)
Electric field	2	62.95	31.48	90.23
Rotating speed	2	0.28	0.14	0.40
Heating temperature	2	6.28	3.14	9.00
Heating time	2	0.26	0.13	0.37
Total	8	69.77	34.89	100.00
